# SARS-CoV-2 BA.2.86 (“Pirola”): Is it Pi or Just Another Omicron Sublineage?

**DOI:** 10.3390/vaccines11111634

**Published:** 2023-10-25

**Authors:** Daniele Focosi, Pietro Giorgio Spezia, Fabrizio Maggi

**Affiliations:** 1 North-Western Tuscany Blood Bank, Pisa University Hospital, 56124 Pisa, Italy; 2 National Institute for Infectious Diseases “Lazzaro Spallanzani” IRCCS, 00149 Rome, Italy; pietro.spezia@inmi.it (P.G.S.); fabrizio.maggi@inmi.it (F.M.)

The SARS-CoV-2 sublineage BA.2.86 was designated on 17 August 2023 by the PANGO Network. At that time, only four sequences were detected, but the lineage was considered worthy of designation because of the unusually high number (34) of mutations within the Spike protein and its detection across different continents during a much reduced genomic surveillance. On 18 August 2023, it was labeled by the WHO as a “variant under monitoring (VUM)” and by the UKHSA as “V-23AUG-01”. It was soon nicknamed “Pirola” on social media to facilitate communication. At that time, much public attention was focused on the expanding EG.5.1 (“Eris”) sublineage and the so-called “FLip” sublineages harboring the S:L455F+F456L pair. On 5 September 2023, the BA.2.86.1 sublineage was designated by the PANGO Network, harboring the additional ORF1a:K1973R mutation and the C12815T SNP, and the JN.1 descendant harboring S:L455S was designated on September 29 2023. JN.2 (ORF1a:Y621C), JN.3 (ORF1a:T2087I), BA.2.86.2 (ORF7a:E22D), and BA.2.86.3 (C222T, C1960T, T12755C), as well as the descendant of the latter JQ.1 (S:T95I), were designated on 12 October 2023.

Compared to the BA.2 reference strain (which was almost extinct in Summer 2023), the main Spike mutations in BA.2.86 are the ins_16MPLF insertion (an antibody escape supersite within the N-terminal domain), the cyclically recurring Δ69-70 deletion [1], the ACE2 receptor affinity-enhancing R403K mutation, the unique 2-nucleotide mutation L452W (reminiscent of the well-known L452R mutation), the unique ΔV483 deletion, and the 2-nucleotide mutation A484K. Global ACE2 affinity resulting from the combination of individual affinity-enhancing (R403K, N460K, R493Q reversion) and affinity-decreasing mutations (delV483, F486P) was predicted to be very high based on deep mutational scanning [2,3], and was confirmed in vitro [4] as higher than XBB.1.5, EG.5.1, and the HK.3 “FLip”s (an emerging group of sublineages with the S:L455F+S:F456L mutation combo) [5].

Importantly, in antigenic cartography, the serological distance between BA.2.86 and wild-type SARS-CoV-2 was found to be longer than from SARS-CoV-1 (when estimated in mice immunized by Spike mRNA [5]) and almost as long as for the “FLip”s in wild-type (wt)+BA.5 bivalent-vaccinated, wt-monovalent-vaccinated, and XBB.1.5-wave-infected humans [6]. Such distance is higher for JN.1 compared to BA.2.86. Additionally, divergence from BA.2 was as long as for XBB.1.5 in wt+BA.5 bivalent-vaccinated or BA.2- or XBB.1.5-breakthrough-infected humans [4]. This distance led some scientists to suggest naming BA.2.86 as “Pi” (the next available Greek letter in the WHO variant nomenclature).

At the time of writing, 1047 BA.2.86 sequences have been counted in just 10 weeks across all continents [7], with many countries reporting detections from surveillance of wastewater samples: notoriety bias and increased sequencing rates represent potential biases. An epidemiological model based on Danish data only estimated the *R*_e_ to be 1.1–1.5 relative to XBB.1.5 [8].

Using pseudoviruses, BA.2.86 was found to be less infectious than XBB.1.5 and EG.5.1 in HOS-ACE2/TMPRSS2 (a fibroblast and epithelial-like human osteosarcoma cell line transduced with both human ACE2 and TMPRSS2) [8], HEK293T-ACE2 [5], and Vero cells [5]. When using live authentic BA.2.86 instead, the focus area and the replication kinetics were identical to XBB.1.5 [9], as expected from the cell-entry-enhancing P1143L mutation. Interestingly, authentic BA.2.86 established a primary infection from clinical sample material in human-derived cell lines CaLu-3 and iGROV-1, but not African Green Monkey-kidney-derived cell lines Vero E6, Vero/hSLAM, and VeroE6 expressing ACE-2 and TMPRSS2 [10].

Contrary to the expectations stemming from BA.2.86 harboring the fusion-enhancing Δ69-70 deletion, BA.2.86 was found to be less fusogenic than XBB.1.5 in HEK-293T and HEK-293T-hACE2 cells [5], but more fusogenic in lung carcinoma CaLu cells [6]. The Ravi Gupta lab in Cambridge further reported on Twitter/X that the increased fusogenicity of BA.2.86 (the highest among the Omicron lineages, albeit much lower than Delta) was dependent on Δ69-70 deletion.

Most of humanity has currently achieved hybrid immunity via multiple exposures to vaccines and SARS-CoV-2. Data on the capacity of breakthrough-infected (BTI) subjects to neutralize BA.2.86 have been reported by multiple groups. In general, either BA.2 BT alone [4], BF.7/BA.5 BTI alone [10,11,12], or XBB BTI alone [4,5,6,8,10,11] were not enough to elicit protective titers, but BA.5/BF.7+XBB BTI [5] or (monovalent or bivalent) vaccination plus BA.1/BA.4/BA.5 BTI [9] or XBB.1.5 BTI [13] raised titers, in line with the heterologous immunity theory formerly validated for COVID-19-convalescent plasma [14,15]. Regardless of vaccine status, pre-Omicron [9], BA.1 [9], or pre-XBB [16] BTI sera neutralized poorly, but contemporary sera instead had high titers [16]. All of these studies were run using pseudoviruses, except for three which employed live authentic BA.2.86 [9,10,13].

Immune escape from vaccine-elicited neutralizing antibodies has been investigated in small cohorts (about 20 per group) of recipients of monovalent mRNA vaccines (three [6,8] or four doses [8]), wt+BA.1 bivalent mRNA vaccines [8], wt+BA.5 bivalent mRNA vaccines [4,6,8,10,17], the tetravalent subunit vaccine SCTV01E [11], and the inactivated protein subunit vaccine ZF2001 or ZF2202-A [12]. All of these studies were run with pseudoviruses, except for two on wt+BA.5 which employed live authentic BA.2.86 [10,13].

Data on the efficacy of the upcoming XBB.1.5-based monovalent mRNA vaccines look promising: results showing high neutralizing antibody titers have been prepublished by Moderna (based on its phase II/III clinical trial of mRNA.1273.815 [18]) and press-released by Pfizer (based on immunized mice) [19].

Only two case series of patients infected by BA.2.86 are available to date. In a technical briefing from the UK, a BA.2.86.1 outbreak was reported in a care home in Norfolk, with a 87% attack rate, but only 1 out of 32 cases required hospitalization [20,21]. Denmark described the first 10 cases: 5 had chronic conditions, all received a 3rd vaccine dose 300–600 days previously, and 50% had a documented previous infection. Again, symptoms reported were similar to those seen for other variants, including cough, shortness of breath, and fever, and none were severely ill [22]. Overall, there is no specific symptom for BA.2.86 [23].

In vitro models [24] predicted resistance to casirivimab (due to V445H and E484K), imdevimab (due to V445H and N450D), bamlanivimab (due to E484K), cilgavimab (due to V445H, N450D, and L452W), tixagevimab (due to E484K), bebtelovimab (due to V445H), regdanvimab (due to L452W and E484K), and most importantly to sotrovimab (due to K356T). These predictions were largely confirmed by real data with pseudoviruses for casirivimab+imdevimab [5], cilgavimab+tixagevimab [5,8,16], bebtelovimab [5,8,16], and sotrovimab [5,6,8,16], leaving BA.2.86 as an orphan of effective mAbs. This is not novel given the lack of efficacy and deauthorization of all the authorized anti-Spike mAbs since Spring 2023 [25,26], and continues to have relevant implications for frail patients who cannot tolerate small-molecule antivirals.

The BA.2.86 variant is capturing the attention of virologists and health officials for its highly mutated Spike and worldwide spread. Based on 2-month follow up, there is no evidence that the BA.2.86 variant is causing a more severe illness, and it does not seem able to outcompete the currently dominant XBB* sublineages (and in particular the “FLip” members). Nevertheless, ongoing BA.2.86 circulation could facilitate the accumulation of more immune evasive mutations (a possibility granted by its high ACE2 affinity), as it is occurring in JN.1, leading to higher spread. Reassuringly, the novel vaccine formulations seem to still induce neutralizing antibody titers that should protect against severe disease. Monitoring of BA.2.86 in wastewaters could prove expecially informative [27].
